# The Impact of Post-contrast Acute Kidney Injury on In-hospital Mortality After Endovascular Thrombectomy in Patients With Acute Ischemic Stroke

**DOI:** 10.3389/fneur.2021.665614

**Published:** 2021-06-07

**Authors:** Mona Laible, Ekkehart Jenetzky, Markus Alfred Möhlenbruch, Martin Bendszus, Peter Arthur Ringleb, Timolaos Rizos

**Affiliations:** ^1^Department of Neurology, Heidelberg University Hospital, Heidelberg, Germany; ^2^Department of Neurology, University of Ulm, Ulm, Germany; ^3^Faculty of Health/School of Medicine, Witten/Herdecke University, Witten, Germany; ^4^Department for Child and Adolescent Psychiatry, Johannes Gutenberg-University, Mainz, Germany; ^5^Department of Neuroradiology, Heidelberg University Hospital, Heidelberg, Germany

**Keywords:** endovascular thrombectomy, ischemic stroke, post-contrast acute kidney injury, renal impairment, in-hospital mortality

## Abstract

**Background and Purpose:** Clinical outcome and mortality after endovascular thrombectomy (EVT) in patients with ischemic stroke are commonly assessed after 3 months. In patients with acute kidney injury (AKI), unfavorable results for 3-month mortality have been reported. However, data on the in-hospital mortality after EVT in this population are sparse. In the present study, we assessed whether AKI impacts in-hospital and 3-month mortality in patients undergoing EVT.

**Materials and Methods:** From a prospectively recruiting database, consecutive acute ischemic stroke patients receiving EVT between 2010 and 2018 due to acute large vessel occlusion were included. Post-contrast AKI (PC-AKI) was defined as an increase of baseline creatinine of ≥0.5 mg/dL or >25% within 48 h after the first measurement at admission. Adjusting for potential confounders, associations between PC-AKI and mortality after stroke were tested in univariate and multivariate logistic regression models.

**Results:** One thousand one hundred sixty-nine patients were included; 166 of them (14.2%) died during the acute hospital stay. Criteria for PC-AKI were met by 29 patients (2.5%). Presence of PC-AKI was associated with a significantly higher risk of in-hospital mortality in multivariate analysis [odds ratio (OR) = 2.87, 95% confidence interval (CI) = 1.16–7.13, *p* = 0.023]. Furthermore, factors associated with in-hospital mortality encompassed higher age (OR = 1.03, 95% CI = 1.01–1.04, *p* = 0.002), stroke severity (OR = 1.05, 95% CI = 1.03–1.08, *p* < 0.001), symptomatic intracerebral hemorrhage (OR = 3.20, 95% CI = 1.69–6.04, *p* < 0.001), posterior circulation stroke (OR = 2.85, 95% CI = 1.72–4.71, *p* < 0.001), and failed recanalization (OR = 2.00, 95% CI = 1.35–3.00, *p* = 0.001).

**Conclusion:** PC-AKI is rare after EVT but represents an important risk factor for in-hospital mortality and for mortality within 3 months after hospital discharge. Preventing PC-AKI after EVT may represent an important and potentially lifesaving effort in future daily clinical practice.

## Introduction

Mortality after 3 months is an important outcome parameter in most observational and clinical stroke trials, including the most recent studies on endovascular stroke treatment [endovascular thrombectomy (EVT)] (1, 2). Well-established factors known to influence midterm to long-term mortality after EVT encompass older age (3–6), higher stroke severity at admission (3–8), larger infarct size (3, 5), lower reperfusion results (3, 5, 7, 8), symptom onset to groin times of more than 6 h (3), and absence of intravenous thrombolysis (3, 5). In addition, comorbid baseline renal impairment has been identified to be associated with mortality after 3 months (8, 9).

A better knowledge on factors influencing, in particular, early mortality would be of high interest for treatment decisions and for predicting short-term prognosis in these seriously affected patients. Nonetheless, only few studies have evaluated early in-hospital mortality in stroke patients irrespective of recanalization treatments (1, 10–14), and even fewer reports are available with regard to the in-hospital mortality after EVT (7, 15). By now, initial stroke severity and increasing age were reported to represent strong predictors of in-hospital mortality after EVT in one single study (7). This study also included information on kidney function, and a higher in-hospital mortality rate in patients with acute kidney injury (AKI) was reported (7). AKI represents an acute and complex renal function disorder that can be observed in up to 50% of patients treated on intensive care units (16–19). The contrast agents applied for computed tomography angiography (CTA) and cerebral angiography have been discussed to precede post-contrast AKI (PC-AKI) (20).

PC-AKI in ischemic stroke patients undergoing EVT appears to particularly affect patients with preexisting renal dysfunction (7, 21), but the role of acute renal disease for short-term outcome after EVT remains largely unknown. Therefore, we here explored whether PC-AKI after EVT has an impact particularly on in-hospital mortality in acute ischemic stroke patients.

## Patients And Methods

Acute ischemic stroke patients 18 years or older who underwent CTA and subsequent EVT between October 2010 and August 2018 at the Neurological Department of Heidelberg University Hospital were included into a prospective and consecutively recruiting database. Patients were either presented directly to our center or transferred by collaborating smaller hospitals of a regional stroke network. Clinical baseline characteristics of all patients were recorded, including patient's age, the premorbid modified Rankin Scale score (pmRS), stroke severity measured by the National Institutes of Health Stroke Scale (NIHSS) at admission and upon discharge or the last value available, the hospital stay length, and important cardiovascular risk factors, e.g., hypertension, diabetes, hypercholesterolemia, and coronary heart disease. Concordant to previous work (3, 4), the pmRS was dichotomized at a scale of 1 (0–1 and 2–5) to reflect preexisting functional disability. Information of baseline renal impairment, including renal replacement therapy, was extracted from medical records. According to current guidelines (22), we classified estimated glomerular filtration rate (eGFR) values <60 mL/min per 1.73 m^2^ at admission as presence of an abnormality of kidney function and presumed baseline renal impairment. The Alberta Stroke Program Early CT Score (ASPECTS) and posterior circulation (pc-)ASPECTS were calculated for all patients.

For CTA, a standardized dose of 65 mL of iodinated contrast dye iobitridol (Xenetix® 350, 38.39 g/50 mL iobitridol; Guerbet, Sulzbach, Germany) was administered intravenously. For cerebral angiography, iopamidol (Solutrast® 300, 30.62 g/50 mL iopamidol; Bracco Imaging Deutschland GmbH, Germany) was administered in 50-mL steps intra-arterially. An approximate median amount of 215 mL contrast dye per patient was applied. EVT was performed by experienced board-certified neuroradiologists, and recanalization results were recorded in each patient according to the Thrombolysis In Cerebral Infarction (TICI) grading system by the treating neuroradiologist. TICI scores of 0–2a were defined as failed recanalization. A subsequent control computed tomography (CT) or magnetic resonance imaging was performed within 20–36 h or earlier in case of clinical deterioration to assess intracerebral hemorrhage (ICH). Symptomatic ICH (sICH) was defined as an intracranial hemorrhage associated with an increase in the NIHSS score of ≥4 points or leading to death (23). An early neurological deterioration was defined as an increase of the NIHSS score ≥ 4. We also documented whether there were decisions to restrict medical treatment and to follow a best-supportive care strategy during the acute hospital stay.

For the present analysis, we included all patients in which information on renal function before and up to 48 h after EVT was available [creatinine, eGFR]. Following the previous recommendation for reporting contrast-associated AKI, PC-AKI was defined as a >25% increase of the baseline serum creatinine value or an absolute increase of serum creatinine of ≥0.5 mg/dL within 48 h after EVT (20). All patients who received renal replacement therapy prior to stroke defined as chronic hemodialysis or peritoneal dialysis were excluded from final analysis, because for them, assessment of PC-AKI was unreasonable. In-hospital mortality as well as the mRS at 3 months were recorded via chart review or telephone interview. The local ethics committee of the Medical Faculty of Heidelberg University approved the study. Because of the study character, patients' consent was waived.

### Statistical Analysis

SPSS, IBM, version 25.0 (IBM, Armonk, NY), was used for all statistical analyses. The primary binary outcome was in-hospital mortality. Our secondary binary outcomes were 3-month mortality, including only patients who survived the acute hospital stay, the development of PC-AKI, unfavorable functional outcomes (dichotomized at a mRS scale at 3 months of 1 and 2), early neurological deterioration, and sICH. We performed a separate analysis of in-hospital mortality and 3-month mortality for anterior circulation stroke and after excluding patients with baseline renal impairment. Differences among groups regarding were compared using the Mann–Whitney *U*-test, the independent *t*-test, and the χ^2^ test according to the scale of variables. Repeated measurements of eGFR values were compared with the paired samples *t*-test. For multivariable logistic regression analyses, we selected variables formerly described to be associated with mortality and PC-AKI (2, 4, 7, 24, 25).

To evaluate whether the relationship between the PC-AKI and in-hospital mortality may depend on other important variables reported to be associated with mortality in patients undergoing EVT (age, NIHSS, pmRS > 1, baseline renal impairment, posterior circulation stroke, failed recanalization, and sICH), a moderation analysis was performed by using the PROCESS macro version 3.5 for SPSS, model 1 according to Hayes (26). All tests were two-sided, and *p* ≤ 0.05 was considered significant.

## Results

### Characteristics of Included Patients

A total of 1,205 patients who were treated with EVT due to acute ischemic stroke were included into the database. Of these, 36 patients had to be excluded because of missing creatinine values at baseline or within 48 h (*n* = 24, 2.0%) or because of long-term renal replacement therapy (*n* = 12, 1.0%), respectively. Hence, 1,169 patients entered the final analysis. As summarized in Table 1, the median age of included patients was 76 years [interquartile range (IQR) = 66–82 years], and the majority were female (*n* = 608, 52.0%).

**Table 1:** Clinical characteristics concerning in-hospital mortality vs. survivors of the acute hospital stay.

	**All**	**In-hospital mortality**	**Survivors of acute hospital stay**	***p*-value**
	**(*n* = 1,169)**	**(*n* = 166)**	**(*n* = 1,003)**	
Female sex, *n* (%)	608 (52.0)	80 (48.2)	528 (52.6)	0.288^#^
Age, median (IQR)	76 (66–82)	79 (71–83)	75 (65–82)	**0.001^§^**
Hospital stay length (days), median (IQR), *n* = 1,169	4 (3–7)	—	5 (4–7)	—
**Functional impairment**				
NIHSS score at admission, median (IQR), *n* = 1,169	17 (11–21)	20 (14–26)	16 (11–21)	**<0.001^§^**
pmRS, median, IQR, *n* = 1,167	1 (0–2)	1 (0–2)	1 (0–2)	0.055^§^
Preexisting functional impairment (pmRS > 1), *n* (%), *n* = 1,167	416 (35.6)	67 (40.4)	349 (34.8)	0.151^#^
3-month mRS, median, IQR, *n* = 1,002	—	—	3 (1–4)	—
Switch to best supportive care, *n* (%), *n* = 1,169	150 (12.8)	142 (85.5)	8 (0.8)	**<0.001^§^**
**Comorbidities**				
Hypertension, *n* (%), *n* = 1,136	878 (75.1)	126 (75.9)	752 (75.0)	0.879^#^
Diabetes, *n* (%), *n* = 1,138	259 (22.2)	44 (26.5)	215 (21.4)	0.179^#^
Hypercholesterolemia, *n* (%), *n* = 1,158	392 (33.5)	49 (29.5)	343 (34.2)	0.323^#^
Coronary heart disease, *n* (%), *n* = 1,147	315 (26.9)	54 (32.5)	261 (26.0)	0.090^#^
AF, *n* (%), *n* = 1,142	569 (48.7)	82 (49.4)	487 (48.6)	0.961^#^
Previous stroke, *n* (%), *n* = 1,134	243 (20.8)	42 (25.3)	201 (20.0)	0.145^#^
BP systolic, mean, SD, *n* = 1,037	143 (72)	158 (27)	160 (27)	0.577^*^
**Medical treatment**				
Antiplatelets at baseline, *n* (%), *n* = 1,136	389 (33.3)	69 (41.6)	320 (31.9)	**0.006^#^**
OAC at baseline, *n* (%), *n* = 1,125	210 (18.0)	25 (15.1)	185 (18.4)	0.342^#^
Statin at baseline, *n* (%), *n* = 1,094	330 (28.2)	38 (22.9)	292 (29.1)	0.182^#^
Additional thrombolysis, *n* (%), *n* = 1,169	697 (59.6)	84 (50.6)	613 (61.1)	**0.011**^**#**^
**Renal function**				
Baseline creatinine, mean (SD), *n* = 1,169	1.01 (0.38)	0.98 (0.44)	1.00 (0.37)	0.016^*^
Baseline eGFR, mean (SD), *n* = 1,169	70.4 (29.0)	65.8 (22.8)	71.2 (29.9)	0.026^*^
Baseline renal impairment (eGFR <60 at admission), *n* (%), *n* = 1,169	379 (32.4)	64 (38.6)	315 (31.4)	0.068^#^
PC-AKI, *n* (%), *n* = 1,169	29 (2.5)	8 (4.8)	21 (2.1)	0.037^#^
**Neuroradiological parameters**				
Anterior circulation stroke, *n* (%), *n* = 1,169	1,053 (90.1)	127 (76.5)	926 (92.3)	**<0.001^#^**
Posterior circulation stroke, *n* (%), *n* = 1,169	116 (9.9)	39 (23.5)	77 (7.7)	
***Vessel occlusion**, ***n*****=1,169***				
MCA, *n* (%)	652 (55.8)	53 (31.9)	599 (59.7)	**<0.001^#^**
Carotid-T, *n* (%)	199 (17.1)	35 (21.1)	164 (16.4)	
ICA and MCA, *n* (%)	130 (11.1)	24 (14.5)	106 (10.6)	
ICA, *n* (%)	55 (4.7)	10 (6.0)	45 (4.5)	
BA, *n* (%)	111 (9.5)	37 (22.3)	74 (7.4)	
VA, *n* (%)	2 (0.2)	1 (0.6)	1 (0.1)	
Other, *n* (%)	20 (1.7)	6 (3.6)	14 (1.4)	
ASPECTS, median, IQR, *n* = 1,093	9 (7–10)	9 (7–10)	9 (7–10)	0.364^§^
TICI 2b−3 recanalization, *n* (%), *n* = 1,169	938 (80.2)	117 (70.5)	822 (81.9)	**0.001^#^**
Failed recanalization (TICI 0–2a), *n* (%), *n* = 1,169	231 (19.8)	49 (29.5)	182 (18.1)	
Any ICH after treatment, *n* (%), *n* = 1,132	355 (30.4)	55 (33.1)	300 (29.9)	0.403^#^
sICH after treatment, *n* (%), *n* = 1,132	55 (4.7)	17 (10.2)	38 (3.8)	**<0.001^#^**

*IQR, interquartile range; SD, standard deviation; (p)mRS, (premorbid) modified Rankin Scale; NIHSS, National Institutes of Health Stroke Scale; BP, blood pressure; OAC, oral anticoagulation; eGFR, estimated glomerular filtration rate; PC-AKI, postcontrast acute kidney injury; MCA, middle cerebral artery; ICA, internal cerebral artery; BA, basilar artery; VA, vertebral artery; ASPECTS, Alberta Stroke Program Early CT Score (restricted to anterior circulation stroke patients); TICI, Thrombolysis In Cerebral Infarction; (s)ICH, (symptomatic) interacerebral hemorrhage*.

* *Unpaired t-test*,

§ *Mann–Whitney U-test*,

# *χ^2^-test. P ≤ 0.5 are displayed in bold font*.

The mean initial NIHSS score was 17 (IQR = 11–21). A total of 1,053 patients (90.1%) suffered an anterior circulation stroke. As shown in Table 1, in 384 patients (36.4%), occlusions of the internal carotid artery (ICA), including carotid-T, were detected. One hundred thirty of these 384 patients (33.9%) suffered both ICA and middle cerebral artery (MCA) occlusions. Furthermore, 652 patients (61.9%) suffered isolated occlusions of the MCA (M1 or M2 segment); 17 patients (1.6%) had other anterior circulation vessel occlusions. Posterior circulation strokes were present in 116 patients (9.9%); the majority of these (*n* = 111, 95.7%) occurred because of occlusions of the basilar artery. The median ASPECTS was 9 (IQR = 7–10).

Most patients had no or no relevant disabilities before onset of stroke (pmRS of 0 or 1: *n* = 751, 64.2%). A total of 206 patients (17.6%) had a pmRS of 2; 166 had a pmRS of 3 (14.2%). Moderately severe disability before stroke (pmRS 4) was present in 3.3% of patients (*n* = 39), and in 0.4% (*n* = 5), severe disability (pmRS 5) was present.

#### Renal Function

At admission, in 32.4% (*n* = 379 patients), an eGFR <60 mL/min per 1.73 m^2^ was observed, indicating baseline renal impairment. The mean initial eGFR was 70.4 ± 29.0 mL/min per 1.73 m^2^, and eGFR follow-up within 48 h was in mean 76.8 ± 24.0 mL/min per 1.73 m^2^ (*p* < 0.001). Within 48 h, an increase of creatinine of ≥0.5 mg/dL or an increase of baseline serum creatinine of >25% was detected in 29 patients (2.5%), thus fulfilling criteria of PC-AKI after EVT.

Age of PC-AKI patients [median age = 72 years (IQR = 58–82 years)] did not differ from non–PC-AKI patients [median age = 76 years (IQR = 66–82 years), *p* = 0.342; cf. Supplementary Table 1]. Also, the prevalence of cardiovascular risk factors among patients with and without PC-AKI did not differ (cf. Supplementary Table 1). Overall, only 10% of PC-AKI patients (*n* = 3) had already baseline renal impairment at admission (e.g., GFR <60 mL/min per 1.73 m^2^). These were 3 of a total of 379 patients with baseline renal impairment who developed PC-AKI (0.8%) As presented in the Supplementary Table 1, PC-AKI patients had a median length of stay of 5 days (3–13 days) vs. 4 days (3–7 days) in patients without PC-AKI (*p* = 0.253). There was no significant association between PC-AKI and the duration of inpatient treatment in univariable analysis. In multivariable logistic regression analysis, as shown in Supplementary Table 2, PC-AKI patients were significantly less likely to present with baseline renal impairment compared to patients who did not develop PC-AKI [odds ratio (OR), 0.23, 95% confidence interval (CI) = 0.07–0.76, *p* = 0.016]. Patients who had received additional thrombolysis seemed to be at a significantly higher risk to develop PC-AKI (OR = 3.47, 95% CI = 1.56–7.71, *p* = 0.002). Factors that may have contributed to AKI development in addition to contrast agents are shown in the Supplementary Table 3. As most frequent competing reasons for PC-AKI, we identified pneumonia and/or sepsis in 35% (*n* = 10).

#### In-hospital Mortality

During the acute hospital stay, 166 patients undergoing EVT died (14.2%; Table 1). The median duration between admission and death was 2 days (IQR = 2–4 days). These patients were older than patients surviving the acute post-EVT phase (*p* = 0.001) and had more severe strokes as measured by the NIHSS score at admission (*p* < 0.001), and they more often suffered posterior circulation strokes (*p* < 0.001). Clinical and treatment-specific variables in patients with in-hospital mortality and patients surviving the acute hospital stay are given in Table 1, as are the results of univariate correlation analyses.

In multivariable analysis (Table 2), the presence of PC-AKI was independently associated with a higher risk of in-hospital mortality (OR = 2.87, 95% CI = 1.16–7.13, *p* = 0.023). Expectedly, further factors independently associated with in-hospital mortality encompassed posterior circulation stroke (OR = 2.85, 95% CI = 1.72–4.71, *p* < 0.001), sICH (OR = 3.20, 95% CI = 1.69–6.04, *p* < 0.001), and failed recanalization procedures (OR = 2.00, 95% CI = 1.35–3.00, *p* = 0.001). Moreover, increasing age (OR = 1.03, 95% CI = 1.01–1.04, *p* = 0.002) and higher stroke severity were associated with a higher risk of in-hospital mortality in multivariable analysis (OR = 1.05, 95% CI = 1.03–1.08, *p* < 0.001). In-hospital deaths without PC-AKI received significantly more often additional thrombolysis compared to those with PC-AKI (52.5 vs. 25%, *p* = 0.027), c.f. Supplementary Table 4.

**Table 2:** Multivariable logistic regression analysis for in-hospital mortality.

	**In-hospital mortality**, ***n*** **=** **166 of 1,169 patients**		
	**Multivariable logistic regression analysis**		
	**OR**	**95% CI**	***P***
Age (per year increasing)	1.03	1.01–1.04	**0.002**
NIHSS score at admission (per point increasing)	1.05	1.03–1.08	**<0.001**
Preexisting functional impairment (pmRS > 1 vs. ≤ 1)	0.86	0.59–1.25	0.415
Baseline renal impairment (eGFR <60 vs. ≥60 at admission)	1.23	0.84–1.80	0.286
PC-AKI (vs. no PC-AKI)	2.87	1.16–7.13	**0.023**
Posterior circulation stroke (vs. anterior circulation stroke)	2.85	1.72–4.71	**<0.001**
Failed recanalization (TICI 0–2a vs. 2b−3)	2.00	1.35–3.00	**0.001**
sICH vs. no sICH	3.20	1.69–6.04	**<0.001**

*NIHSS, National Institutes of Health Stroke Scale; eGFR, estimated glomerular filtration rate; PC-AKI, postcontrast acute kidney injury; TICI, Thrombolysis In Cerebral Infarction; sICH, symptomatic intracerebral hemorrhage. P ≤ 0.5 are displayed in bold font*.

Moderation analysis indicated that there was no moderating effect of age (*p* = 0.167), stroke severity (NIHSS, *p* = 0.945), preexisting functional impairment (*p* = 0.484), baseline renal impairment (*p* = 0.985), posterior circulation stroke (*p* = 0.569), failed recanalization (*p* = 0.400), or sICH (*p* = 0.987) on the relationship between PC-AKI and in-hospital mortality.

#### Mortality at 3 Months

For the analysis of mortality at 3 months, we subsequently included patients who survived the acute hospital stay only (*n* = 1,003). Three-month outcome data in these patients were available in 99.8% (*n* = 1,001); mortality at 3 months was 14.6% (146/1,001).

A considerably higher 3-month mortality rate was observed in case of PC-AKI compared to patients without PC-AKI (33.3% vs. 14.2%; *p* = 0.014, Supplementary Table 1). Results of univariate correlation analyses between patients who died between hospital discharge and follow-up, and those surviving 3 months are summarized in Supplementary Table 5.

In multivariable logistic regression (Table 3), again, patients with PC-AKI had a substantially higher risk of death (OR = 3.70, 95% CI = 1.19–11.53, *p* = 0.024). In addition, increasing age (OR = 1.04, 95% CI = 1.02–1.06, *p* < 0.001), stroke severity (OR = 1.08, 95% CI = 1.05–1.11, *p* < 0.001), preexisting functional impairment (OR = 2.44, 95% CI = 1.64–3.63, *p* < 0.001), failed recanalization (OR = 2.69, 95% CI = 1.74–4.17, *p* < 0.001), and sICH (OR = 2.94, 95% CI = 1.30–6.65, *p* = 0.009) were significant risk factors for mortality within 3 months.

**Table 3:** Multivariable logistic regression analysis for 3-month mortality.

	**3-month mortality (*****n*** **=** **146, only survivors of the acute hospital stay were considered)**		
	**Multivariable logistic regression analysis**		
	**OR**	**95% CI**	***P***
Age (per year increasing)	1.04	1.02–1.06	**<0.001**
NIHSS score at admission (per point increasing)	1.08	1.05–1.11	**<0.001**
Preexisting functional impairment (pmRS > 1 vs. ≤ 1)	2.44	1.64–3.63	**<0.001**
Baseline renal impairment (eGFR <60 vs. ≥60 at admission)	1.14	0.76–1.71	0.535
PC-AKI (vs. no PC-AKI)	3.70	1.19–11.53	**0.024**
Posterior circulation stroke (vs. anterior circulation stroke)	0.32	0.14–0.76	**0.010**
Failed recanalization (TICI 0–2a vs. 2b−3)	2.69	1.74–4.17	**<0.001**
sICH vs. no sICH	2.94	1.30–6.65	**0.009**

*NIHSS, National Institutes of Health Stroke Scale; pmRS, premorbid modified Rankin Scale; eGFR, estimated glomerular filtration rate; PC-AKI, post-contrast-AKI, TICI, Thrombolysis In Cerebral Infarction; sICH, symptomatic intracerebral hemorrhage. P ≤ 0.5 are displayed in bold font*.

The main results of multivariable regression analyses regarding in-hospital mortality remained unchanged when only patients without baseline renal impairment were considered (c.f. Supplementary Table 6), but PC-AKI no longer was associated with 3-month mortality.

#### Functional Outcome

**Figure 1:**
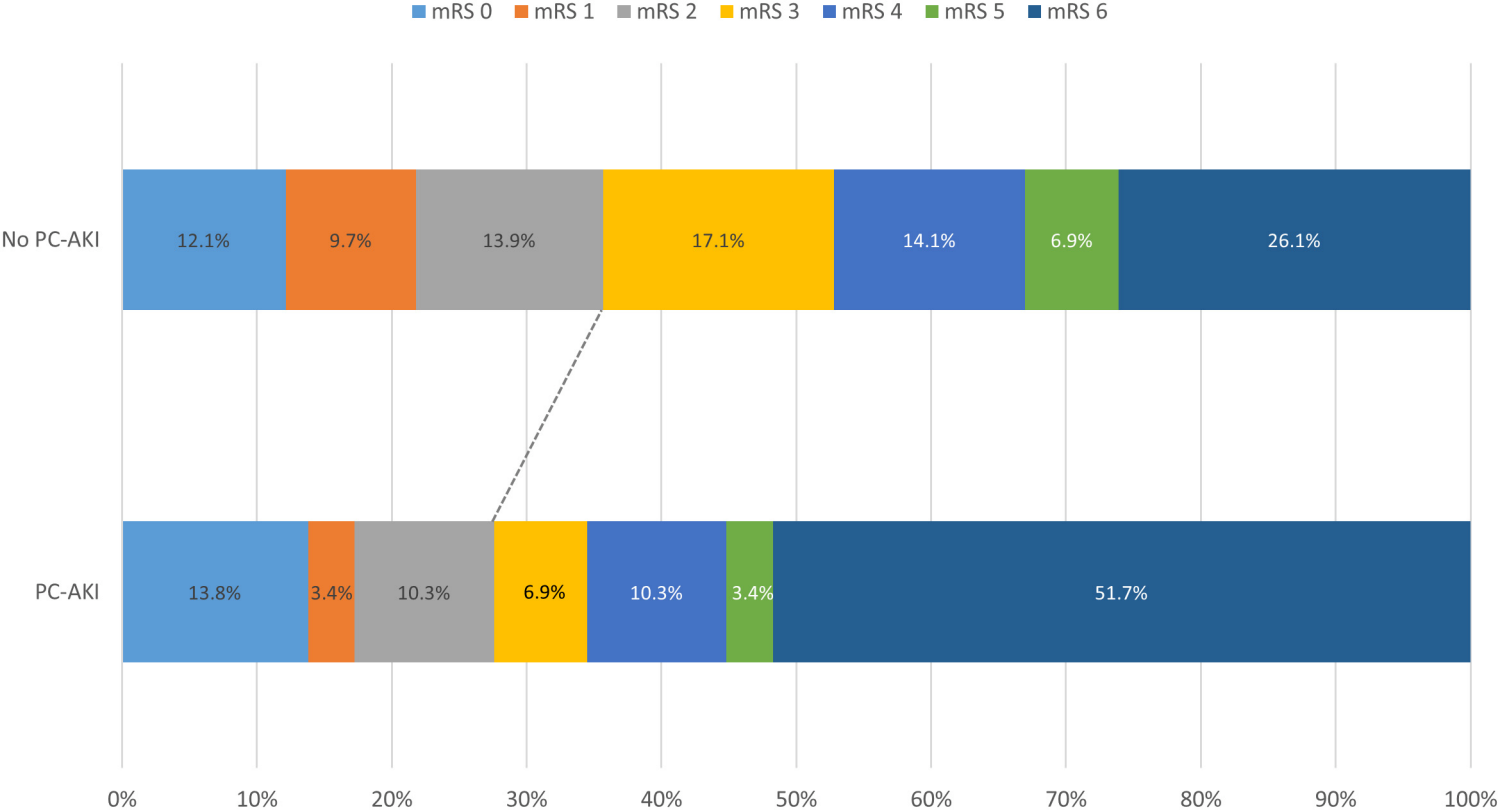
Clinical outcomes of patients with and without PC-AKI. All 1,169 patients were considered. The cutt-off for functional dependence was set at a mRS of >2.

PC-AKI had no impact on the risk of an unfavorable functional outcome (mRS > 1 and mRS > 2), early neurological deterioration, or sICH, as shown in the Supplementary Table 7. As illustrated by Figure 1, the rate of functional dependence (mRS > 2) after 3 months with PC-AKI and without PC-AKI was 72.4 vs. 64.2% and did not differ significantly (*p* = 0.369).

#### Anterior Circulation Stroke

As shown in the Supplementary Table 8, the same factors had a significant impact on 3-month mortality in anterior large vessel occlusion (LVO) alone as it was the case for the whole cohort. These were PC-AKI, increasing age, increasing NIHSS values at admission, a preexisting functional impairment, failed recanalization, and sICH. However, with regard to in-hospital mortality in anterior LVO exclusively, the impact of a PC-AKI on in-house mortality was not significant (c.f. Supplementary Table 8).

## Discussion

The main findings of our study are that PC-AKI after EVT in patients with acute ischemic stroke is strongly associated both with mortality in-hospital and 3 months after hospital discharge.

It is a matter of current discussion whether AKI in hospitalized patients represents a surrogate of general illness or whether its development during the hospital stay is caused by specific medical procedures (27). Previous work suggests that the contrast agent may contribute to PC-AKI in some patients, as creatinine values > 1.6 mg/dL were found to make individuals more prone to develop PC-AKI during hospitalization (28). Other recent reports did not observe associations between amounts of applied contrast agent and a risk of AKI after EVT (7, 21) and after contrast-enhanced CT imaging (29). Besides contrast-induced nephropathy (30), further reported important mechanisms for the development of AKI in acute ischemic stroke patients also encompass hemodynamic changes following blood pressure variations (31), acute tubular lesions (32), intravasal volume deficiency (30), and inflammatory conditions (20, 32). In our cohort, all of these mechanisms may apply to some extent as general anesthesia during EVT in example frequently involves drops in systolic blood pressure <140 mmHg (33) and all patients received contrast dye two times (for CTA and for EVT). In addition, acute infections, i.e., pneumonia or urinary tract infections, are common complications in the post–acute phase after stroke (34).

Compared to previous studies evaluating the incidence of PC-AKI in ischemic stroke patients undergoing EVT (7, 21, 35–37), our overall rate of PC-AKI was within the lower range of previously reported incidences [2.5 vs. 1.5% (35) to 7.3% (7, 21, 36, 37)], although no pre-specified hydration protocol was used. Differences of the AKI incidence may be explained by a varying post-admission observation period of 48 h (35, 36) vs. 72 h (7, 21, 37) and up to 5 days (7) and dissimilar criteria used for AKI detection (7, 21, 35). PC-AKI in ischemic stroke patients who undergo EVT has been reported to particularly affect patients with baseline renal impairment (7, 21). In contrast, and unlike previous studies in which approximately one-third of AKI patients had the preexisting comorbidity of a chronic kidney disease (38, 39), we observed that baseline renal impairment was numerically less common in patients with PC-AKI compared to patients without PC-AKI (10.3 vs. 33.0%). Also, decreasing eGFR values did not correlate with the risk of PC-AKI, as described previously for a level <30 mL/min per 1.73 m^2^ (21), and baseline renal impairment was a risk neither of short-term nor of long-term mortality in multivariate analysis. Possible reasons for this observation are differing triggers underlying AKI development (38) as baseline renal impairment is only one of various known predisposing factors for AKI. In addition, different approaches were used to define baseline renal impairment across studies (7, 21).

On the other hand, in-hospital mortality in our patients with PC-AKI (28%) is comparable to the mortality rate of the report by Weber et al. [20% (7)]. Of note, adjusted for important confounders, PC-AKI more than doubled the risk of in-hospital death in our patients. This corresponds well to the results of the aforementioned study (7). Importantly, the additional moderation analysis performed in the present study did not reveal that PC-AKI and in-hospital mortality depended on other known factors associated with mortality in our large cohort of patients undergoing EVT. This hence indicates that PC-AKI appears to represent an independent risk factor for short-term and midterm mortality after EVT.

Neither the previously proposed PREMISE risk model (10) to assess the risk of early mortality after acute ischemic stroke nor the American Get With The Guidelines Stroke Program mortality score (40) did consider AKI or renal function. On the other hand, the IScore to predict poor functional outcomes early after hospitalization for an acute ischemic stroke included severe kidney dysfunction requiring renal replacement therapy (41). Here, renal dialysis was observed to be independently associated with 30-day mortality risk (41). Adding the findings of the recent study, our data indicate that acute renal dysfunction (i.e., PC-AKI) represents an important but so far often neglected risk factor for early mortality and 3-month mortality after EVT. Therefore, PC-AKI should be considered for future clinical scores to facilitate treatment decisions in this group of frequently seriously affected acute ischemic stroke patients.

The consecutive recruitment of all EVT patients admitted to our high-volume primary stroke center, thorough collection of laboratory parameters, and assessment of in-hospital mortality as well as outcome assessment after 3 months represent obvious strengths of our analysis. Limitations include that, similar to most other stroke registries, stroke volumetry was not performed. Moreover, some potential factors, including exact amounts of contrast dye that may have contributed to PC-AKI, could have been missed, and we did not include data on infectious diseases after EVT. In addition, decisions to limit medical care may have influenced the numbers of in-hospital deaths, and although acute stroke treatments are highly standardized within the regional network, we cannot completely exclude a selection bias.

## Conclusions

To conclude, our results indicate that PC-AKI, although rare, represents an important but so far often neglected risk factor for in-hospital mortality and for mortality within 3 months after hospital discharge in acute ischemic stroke patients undergoing EVT. Hence, preventing AKI in these patients appears to represent an important and potentially lifesaving effort in future daily clinical practice.

## Data Availability Statement

The raw data supporting the conclusions of this article will be made available by the authors, without undue reservation.

## Ethics Statement

The studies involving human participants were reviewed and approved by Ethics Committee of the Medical Faculty of Heidelberg University, Alte Glockengießerei 11/1, 69115 Heidelberg, Germany. Written informed consent for participation was not required for this study in accordance with the national legislation and the institutional requirements.

## Author Contributions

ML: study design, data collection, statistical analysis, data interpretation, manuscript drafting, critical revision, and final approval of the manuscript. EJ: statistical analysis, data interpretation, critical review of the manuscript. MM: performance of EVT, data collection, and critical review of the manuscript. MB: supervision and performance of EVT and data collection, critical review of the manuscript. PR: data collection, critical review of the manuscript. TR: concept and study design, statistical analysis, manuscript drafting, critical revision, and final approval of the manuscript. All authors contributed to the article and approved the submitted version.

## Conflict of Interest

EJ receives funding of the German Federal Ministry of Education and Research (BMBF) and the German Research Foundation (DFG), outside the submitted work. MM reports the following conflicts of interest, unrelated to the submitted work: Consultancy: Medtronic^*^, MicroVention^*^, Stryker Neurovascular^*^, phenox^*^; Grants/Grants Pending: Balt^*^, MicroVention^*^; Payment for Lectures Including Service on Speakers Bureaus: Medtronic^*^, MicroVention^*^, Stryker Neurovascular^*^. ^*^Money paid to the institution. MB reports personal fees from Boehringer Ingelheim, grants and personal fees from Novartis, grants from SIemens, personal fees from Merck, personal fees from Bayer, grants and personal fees from Guerbet, grants from Hopp Foundation, grants from DFG, grants from European Union, grants from Stryker, personal fees from Teva, personal fees from BBraun, personal fees from Vascular Dynamics, personal fees from Grifols, personal fees from Neuroscios, outside the submitted work. PR received honoraria from Bayer, Boehringer Ingelheim, Daichi Sankyo and Pfizer, outside of present work. TR received consulting honoraria, speakers' honoraria and travel support from Bristol-Myers Squibb/Pfizer, Boehringer-Ingelheim, Bayer HealthCare, and DaichiiSankyo, outside of present work. The remaining author declares that the research was conducted in the absence of any commercial or financial relationships that could be construed as a potential conflict of interest.
