# Supplementary material for: The Impact of Post-contrast Acute Kidney Injury on In-hospital Mortality After Endovascular Thrombectomy in Patients With Acute Ischemic Stroke
Source: Front Neurol. 2021 Jun 7;12:665614. doi: 10.3389/fneur.2021.665614 (PMC8215575; doi:10.3389/fneur.2021.665614)
Supplement: Supplementary file 1 [file Table_1.pdf]

	**All (n=1169)**	**PC-AKI (n=29)**	**No PC-AKI (n=1140)**	**p-value**
Female sex, N (%),	608 (52.0)	17 (58.6)	591 (51.8)	0.471[*]
Age, median (IQR)	76 (66-82)	72 (58-82)	76 (66-82)	0.342[§]
Hospital stay length	4 (3-7)	5 (3-13)	4 (3-7)	0.253[§]
**Functional impairment**				
NIHSS at admission, median (IQR), n=1169	17 (11-21)	16 (10-22)	17 (11-21)	0.677[§]
pmRS, median, IQR	1 (0-2)	1 (0-3)	1 (0-2)	0.303[§]
Preexisting functional impairment (pmRS>1) N (%), n=1169	416 (35.6)	12 (41.4)	404 (35.4)	0.514[#]
3-month mRS, median, IQR, n=1002	3 (2-6)	5 (2-6)	3 (2-6)	0.150[§]
In-hospital mortality, N (%), n=1169	165 (14.1)	7 (24.1)	158 (13.9)	0.116[#]
3-months mortality (for survivors of the acute hospital treatment), N (%), n=1002	147/1002 (14.6)	8/22 (36.4)	139/980 (14.2)	**0.004**[#]
**Comorbidities**				
Hypertension, N (%), n=1136	878 (75.1)	23 (79.3)	855 (74.9)	0.792[#]
Diabetes, N (%), n=1138	259 (22.2)	9 (31.0)	250 (21.9)	0.282[#]
Hypercholesterolemia, N (%), n=1158	392 (33.5)	9 (31.0)	383 (33.6)	0.745[#]
Coronary heart disease, N (%), n=1147	315 (26.9)	5 (17.2)	310 (27.2)	0.212[#]
**Medical treatment**				
Antiplatelets at baseline, N (%), n=1136	389 (33.3)	9 (31.0)	380 (33.3)	0.813[#]
OAC at baseline, N (%), n=1137	210 (18.0)	3 (10.3)	207 (18.2)	0.274[#]
Statin at baseline, N (%), n=1094	330 (28.2)	7 (24.1)	323 (28.3)	0.546[#]
Additional thrombolysis, N (%), n=1169	697 (59.6)	9 (31.0)	688 (60.4)	**0.001**[§]
**Renal function**				
Baseline creatinine, mean (SD), n=1169	1.01 (0.38)	0.70 (0.39)	1.02 (0.38)	**0.001**[*]
Baseline eGFR, mean (SD), n=1169	70.4 (29.0)	86.8 (27.5)	70.6 (29.0)	**0.002**[*]
Baseline renal impairment (eGFR<60 at admission), N (%), n=1169	379 (32.4)	3 (10.3)	376 (33.0)	**0.010**[#]
**Neuroradiological parameters**				
Anterior circulation stroke, N (%), n=1169	1053 (90.1)	27 (93.1)	1026 (90.0)	0.581[§]
Posterior circulation stroke, N (%), n=1169	116 (9.9)	2 (6.9)	114 (10.0)	

Vessel occlusion, n=1169				
MCA, N (%)	652 (55.8)	14 (41.4)	638 (56.0)	0.782[#]
Carotid-T, N (%)	199 (17.1)	5 (17.2)	194 (17.0)	
ICA and MCA, N (%)	130 (11.1)	5 (17.2)	125 (11.0)	
ICA, N (%)	55 (4.7)	2 (6.9)	53 (4.7)	
BA, N (%)	111 (9.5)	2 (6.9)	109 (9.6)	
VA, N (%)	2 (0.2)	0 (0)	2 (0.2)	
other, N (%)	20 (1.7)	1 (3.4)	19 (1.7)	
ASPECTS score, median, IQR, n=1093	9 (7-10)	9 (8-10)	9 (7-10)	0.451[*]
TICI 2b-3 recanalization, N (%), n=1169	938 (80.2)	18 (62.1)	918 (80.5)	0.123[§]
Failed recanalization (TICI 0-2a), N (%), n=1169	231 (19.8)	9 (31.0)	222 (19.5)	
Any ICH after treatment, N (%), n=1132	355 (30.4)	9 (31.0)	346 (30.4)	0.937[§]
sICH after treatment, N (%), n=1132	55 (4.7)	2 (6.9)	53 (4.6)	0.569[§]

Supplementary Table 1: Baseline and treatment characteristics of patients with and without post-contrast acute kidney injury

IQR, interquartile range; SD, standard deviation; (p)mRS, (premorbid) modified Rankin Scale; NIHSS, National Institutes of Health Stroke Scale; BP, blood pressure; OAC oral anticoagulation; eGFR, estimated glomerular filtration rate; PC-AKI, post-contrast acute kidney injury; MCA, middle cerebral artery; ICA, internal cerebral artery; BA, basilar artery; VA, vertebral artery; ASPECTS, Alberta Stroke Program Early CT Score (restricted to anterior circulation stroke patients); TICI, Thrombolysis In Cerebral Infarction; (s)ICH, (symptomatic) interacerebral hemorrhage. *unpaired t-test, § Mann Whitney U test, # chi square test. P-values ≤0.5 are displayed in bold.
