# Supplementary material for: The Impact of Post-contrast Acute Kidney Injury on In-hospital Mortality After Endovascular Thrombectomy in Patients With Acute Ischemic Stroke
Source: Front Neurol. 2021 Jun 7;12:665614. doi: 10.3389/fneur.2021.665614 (PMC8215575; doi:10.3389/fneur.2021.665614)
Supplement: Supplementary file 2 [file Table_2.pdf]

	PC-AKI within 48 hours, n=29					
	Univariable logistic regression analysis			*Multivariable logistic regression analysis*		
	OR	95%-CI	*P*	OR	95%-CI	*P*
Age (per year increasing)	0.99	0.96-1.01	0.269	-	-	-
Female sex (vs. male sex)	0.76	0.36-1.61	0.472	-	-	-
NIHSS at admission (per point increasing)	0.99	0.94-1.03	0.508	-	-	-
Hypertension	1.13	0.46-2.81	0.792	-	-	-
Diabetes	1.55	0.70-3.44	0.285	-	-	-
Coronary heart disease	0.54	0.21-1.44	0.218			
Additional contrast administration (vs. no additional contrast administration)	1.23	0.29-5.26	0.785	-	-	-
Baseline renal impairment (eGFR<60 vs. ≥60 at admission)	0.23	0.07-0.78	**0.018**	0.23	0.07-0.76	**0.016**
Failed recanalization (TICI 0-2a vs. 2b-3)	1.86	0.84-4.14	0.128	-	-	-
Additional thrombolysis (vs. EVT alone)	3.38	1.53-7.50	**0.003**	3.47	1.56-7.71	**0.002**
Posterior circulation stroke (vs. anterior circulation stroke)	0.67	0.16-2.84	0.583	-	-	-
Systolic BP at admission, each mmHg (each mmHg increasing)	1.003	0.99-1.02	0.720	-	-	-

Supplementary Table 2: Uni- and multivariable logistic regression analysis for post-contrast acute kidney injury

PC-AKI, post-contrast-AKI; NIHSS, National Institutes of Health Stroke Scale; eGFR, estimated glomerular filtration rate (mL/min/1.73 m^2); TICI, Thrombolysis In Cerebral Infarction; EVT, endovascular thrombectomy; BP, blood pressure. P-values ≤0.5 are displayed in bold.
