# Supplementary material for: The Impact of Post-contrast Acute Kidney Injury on In-hospital Mortality After Endovascular Thrombectomy in Patients With Acute Ischemic Stroke
Source: Front Neurol. 2021 Jun 7;12:665614. doi: 10.3389/fneur.2021.665614 (PMC8215575; doi:10.3389/fneur.2021.665614)
Supplement: Supplementary file 3 [file Table_3.pdf]

| | All PC-AKI patients (N=29) | PC-AKI patients surviving the acute hospital stay (N=22) |
|---|---|---|
| Pneumonia/sepsis, N (%) | 10 (35) | 8 (36) |
| Acute or decompensated chronic heart failure, N (%) | 6 (11) | 4 (18) |
| Acute urinary tract infection, N (%) | 1 (3) | 1 (5) |
| Acute hypertensive crisis, N (%) | 1 (3) | 1 (5) |
| Ongoing chemotherapy with vinorelbine (for alveolar cellular carcinoma), N (%) | 1 (3) | 1 (5) |
| Cardiopulmonal reanimation, catecholamine therapy, N (%) | 1 (3) | - |
| No identifyable competing trigger for PC-AKI, N (%) | 9 (31) | 7 (32) |

**Supplementary Table 3: Clinical complications and circumstances that may represent a risk for post-contrast acute kidney injury beyond the possibility of a causal relationship with contrast dye**

PC-AKI, post-contrast acute kidney injury.
