# Supplementary material for: The Impact of Post-contrast Acute Kidney Injury on In-hospital Mortality After Endovascular Thrombectomy in Patients With Acute Ischemic Stroke
Source: Front Neurol. 2021 Jun 7;12:665614. doi: 10.3389/fneur.2021.665614 (PMC8215575; doi:10.3389/fneur.2021.665614)
Supplement: Supplementary file 4 [file Table_4.pdf]

	All in-hospital deaths (n=166)	In-hospital deaths with PC-AKI (n=8)	In-hospital deaths without PC-AKI (n=158)	p-value
Female sex, N (%)	80 (48.2)	6 (75.0)	74 (46.8)	0.120[#]
Age, median (IQR)	79 (71-83)	81 (74-88)	78 (70-83)	0.229[§]
**Functional impairment**				
NIHSS at admission, median (IQR), n=166	20 (14-26)	20 (12-25)	20 (14-27)	0.827[§]
pmRS, median, IQR, n=153	1 (0-2)	2 (0-3)	1 (0-2)	0.400[§]
Preexisting functional impairment (pmRS>1), N (%), n=153	67 (40.4)	4 (50)	63 (39.9)	0.569[#]
Switch to best supportive care, N (%), n=158	142 (85.5)	5 (63)	137 (86.7)	0.098[§]
**Comorbidities**				
Hypertension, N (%), n=156	126 (75.9)	5 (63)	121 (76.6)	0.325[#]
Diabetes, N (%), n=156	44 (26.5)	1 (13)	43 (27.2)	0.348[#]
Hypercholesterolemia, N (%), n=153	49 (29.5)	2 (25)	47 (29.7)	0.732[#]
Coronary heart disease, N (%), n=156	54 (32.5)	4 (50)	50 (31.6)	0.292[#]
AF, N (%), n=156	82 (49.4)	6 (75)	76 (48.1)	0.147[#]
Previous stroke, N (%), n=155	42 (25.3)	0 (0)	42 (26.6)	0.087[#]
BP systolic, median, IQR, n=126	159 (139-176)	150 (130-157)	159 (140-176)	0.245[*]
**Medical treatment**				
Antiplatelets at baseline, N (%), n=150	69 (41.6)	3 (38)	66 (41.8)	0.953[#]
OAC at baseline, N (%), n=150	25 (15.1)	1 (13)	24 (15.2)	0.472[#]
Statin at baseline, N (%), n=142	38 (22.9)	3 (38)	35 (22.2)	0.281[#]
Additional thrombolysis, N (%), n=166	84 (50.6)	1 (25)	83 (52.5)	**0.027**[#]
**Renal function**				
Baseline creatinine, median, IQR, n=166	0.98 (0.78-1.25)	0.70 (0.69-0.81)	1.00 (0.80-1.28)	**0.012**[*]
Baseline eGFR, median, IQR, n=166	66.8 (50.2-82.4)	81 (73-96)	64 (40.5)	**0.043**[*]
Baseline renal impairment (eGFR<60 at admission), N (%), n=166	64 (38.6)	0 (0)	64 (40.5)	**0.022**[#]
**Neuroradiological parameters**				
Anterior circulation stroke, N (%), n=166	127 (76.5)	6 (75)	121 (76.6)	0.918[#]
Posterior circulation stroke, N (%), n=166	39 (23.5)	2 (25)	37 (23.4)	
Vessel occlusion, n=166				

MCA, N (%)	53 (31.9)	4 (50)	49 (31.0)	0.985#
Carotid-T, N (%)	35 (21.1)	1 (13)	34 (21.5)	
ICA and MCA, N (%)	24 (14.5)	0 (0)	24 (15.2)	
ICA, N (%)	10 (6.0)	1 (13)	9 (5.7)	
BA, N (%)	37 (22.3)	2 (25)	35 (22.2)	
VA, N (%)	1 (0.6)	0 (0)	1 (0.6)	
other, N (%)	6 (3.6)	0 (0)	6 (3.8)	
ASPECTS, median, IQR, n=153	9 (7-10)	7 (6-10)	9 (7-10)	0.454§
TICI 2b-3 recanalization, N (%), n=166	117 (70.5)	6 (75)	111 (70.3)	0.774#
Failed recanalization (TICI 0-2a), N (%), n=166	49 (29.5)	2 (25)	47 (29.7)	
Any ICH after treatment, N (%), n=166	55 (33.1)	2 (25)	53 (33.5)	0.616#
sICH after treatment, N (%), n=166	10 (6.0)	0 (0)	10 (10.8)	0.326#

Supplementary Table 4: Differences in clinical characteristics in in-hospital deaths with and without PC-AKI

IQR, interquartile range; (p)mRS, (premorbid) modified Rankin Scale; NIHSS, National Institutes of Health Stroke Scale; BP, blood pressure; OAC oral anticoagulation; eGFR, estimated glomerular filtration rate; PC-AKI, post-contrast acute kidney injury; MCA, middle cerebral artery; ICA, internal cerebral artery; BA, basilar artery; VA, vertebral artery; ASPECTS, Alberta Stroke Program Early CT Score (restricted to anterior circulation stroke patients); TICI, Thrombolysis In Cerebral Infarction; (s)ICH, (symptomatic) interacerebral hemorrhage. *unpaired t-test, § Mann Whitney U test, # chi square test. P-values ≤0.5 are displayed in bold.
