# Supplementary material for: The Impact of Post-contrast Acute Kidney Injury on In-hospital Mortality After Endovascular Thrombectomy in Patients With Acute Ischemic Stroke
Source: Front Neurol. 2021 Jun 7;12:665614. doi: 10.3389/fneur.2021.665614 (PMC8215575; doi:10.3389/fneur.2021.665614)
Supplement: Supplementary file 5 [file Table_5.pdf]

	**All (n=1001)**	**Death within 3 months (n=146)**	**Survivors upon 3 months (n=855)**	**p-value**
Female sex, N (%)	527 (52.6)	85 (58.2)	442 (51.7)	0.169[#]
Age, median (IQR)	75 (65-82)	81 (74-86)	75 (64-81)	**<0.001[§]**
**Functional impairment**				
Admission NIHSS, median, IQR, n=1001	16 (11-21)	21 (16-24)	15 (10-20)	**<0.001[§]**
pmRS, median, IQR, n=1000	1 (0-2)	2 (1-3)	1 (0-2)	**<0.001[§]**
Preexisting functional impairment (pmRS>1), N (%), n=1001	349 (34.9)	86 (58.9)	263 (30.8)	**<0.001[#]**
3-month mRS, median, IQR, n=1001	-	-	3 (1-4)	-
**Comorbidities**				
Hypertension, N (%), n=971	750 (74.9)	124 (84.9)	626 (73.2)	**0.011[#]**
Diabetes, N (%), n=973	215 (21.5)	43 (29.5)	172 (20.1)	**0.021[#]**
Hypercholesterolemia, N (%), n=996	343 (34.2)	49 (33.6)	294 (34.4)	0.903[#]
Coronary heart disease, N (%), n=982	261 (26.1)	53 (36.3)	208 (24.3)	**0.001[#]**
AF, N (%), n=977	485 (48.5)	95 (65.1)	390 (45.6)	**<0.001[#]**
Previous stroke, N (%), n=970	201 (20.1)	43 (29.5)	158 (18.5)	**0.003[#]**
BP systolic, mean, SD, n=904	160 (27)	158 (29)	160 (27)	0.461[*]
**Medical treatment**				
Antiplatelets at baseline, N (%), n=977	320 (32.0)	49 (30.1)	271 (31.7)	0.585[#]
OAC at baseline, N (%), n=966	184 (18.4)	42 (28.8)	142 (16.6)	**0.001[#]**
Statin at baseline, N (%), n=951	292 (29.2)	44 (30.1)	248 (29.0)	0.752[#]
Additional thrombolysis, N (%), n=1001	612 (61.1)	88 (60.3)	524 (61.3)	0.817[#]
**Renal function**				
Baseline creatinine, mean (SD), n=1001	1.00 (0.37)	1.04 (0.41)	0.99 (0.37)	0.146[*]
Baseline GFR, mean (SD), n=1001	71.2 (29.9)	69.1 (56.4)	71.5 (22.4)	0.337[*]
Baseline renal impairment (eGFR<60 at admission), N (%), n=1001	315 (31.5)	59 (40.4)	256 (29.9)	**0.012[#]**
PC-AKI, N (%), n=1001	21 (2.1)	7 (4.8)	14 (1.6)	**0.014[#]**
**Neuroradiological parameters**				
Anterior circulation stroke, N (%), n=1001	924 (92.3)	138 (94.5)	786 (91.9)	0.278[#]

Posterior circulation stroke, N (%), n=1001	77 (7.7)	8 (5.5)	69 (8.1)	
Vessel occlusion, n=1001				
MCA, N (%)	598 (59.7)	79 (54.1)	519 (60.7)	0.174#
Carotid-T, N (%)	163 (16.3)	46 (31.5)	117 (13.6)	
ICA and MCA, N (%)	106 (10.6)	4 (2.7)	102 (11.8)	
ICA, N (%)	45 (4.5)	8 (5.5)	37 (4.3)	
BA, N (%)	74 (7.4)	8 (5.5)	66 (7.7)	
VA, N (%)	1 (0.1)	-	1 (0.1)	
other, N (%)	14 (1.4)	1 (0.7)	13 (1.5)	
ASPECTS score, median, IQR, n=938	9 (7-10)	8 (7-10)	9 (7-10)	**0.016§**
TICI 2b-3 recanalization, N (%), n=886	819 (81.8)	101 (69.2)	718 (84.0)	**<0.001#**
Failed recanalization (TICI 0-2a), N (%), n=886	182 (18.2)	45 (30.8)	137 (15.9)	
Any ICH after treatment, N (%), n=1001	300 (30.0)	52 (35.6)	248 (29.0)	0.107#
sICH after treatment, N (%), n=966	38 (3.8)	11 (7.5)	27 (3.2)	**0.015#**

Supplementary Table 5: Clinical characteristics concerning three-month mortality. All survivors of the acute hospital stay with available three-month outcome data were considered (n=1001).

IQR, interquartile range; SD, standard deviation; (p)mRS, (premorbid) modified Rankin Scale; NIHSS, National Institutes of Health Stroke Scale; BP, blood pressure; OAC oral anticoagulation; eGFR, estimated glomerular filtration rate; PC-AKI, post-contrast acute kidney injury; MCA, middle cerebral artery; ICA, internal cerebral artery; BA, basilar artery; VA, vertebral artery; ASPECTS, Alberta Stroke Program Early CT Score; TICI, Thrombolysis In Cerebral Infarction; (s)ICH, (symptomatic) interacerebral hemorrhage. *unpaired t-test, § Mann Whitney U test, # chi square test. P-values ≤0.5 are displayed in bold.
