# Supplementary material for: The Impact of Post-contrast Acute Kidney Injury on In-hospital Mortality After Endovascular Thrombectomy in Patients With Acute Ischemic Stroke
Source: Front Neurol. 2021 Jun 7;12:665614. doi: 10.3389/fneur.2021.665614 (PMC8215575; doi:10.3389/fneur.2021.665614)
Supplement: Supplementary file 6 [file Table_6.pdf]

	In-hospital mortality, n=166 of 1169 patients			*Three-month mortality (N=147, only survivors of the acute hospital stay were considered)*		
	Multivariable logistic regression analyses					
	OR	95%-CI	*P*	OR	95%-CI	*P*
Age (per year increasing)	1.02	1.01-1.05	**0.016**	1.04	1.02-1.07	**<0.001**
NIHSS at admission (per point increasing)	1.06	1.03-1.09	**<0.001**	1.11	1.07-1.15	**<0.001**
Preexisting functional impairment (pmRS>1 vs. ≤1)	0.79	0.48-1.32	0.368	2.53	1.51-4.23	**<0.001**
PC-AKI (vs. no PC-AKI)	3.63	1.35-9.74	**0.011**	2.38	0.63-8.99	0.201
Posterior circulation stroke (vs. anterior circulation stroke)	3.31	1.82-6.04	**<0.001**	0.36	0.13-1.03	0.056
Failed recanalization (TICI 0-2a vs. 2b-3)	1.73	1.02-2.94	**0.042**	3.12	1.79-5.46	**<0.001**
sICH vs. no sICH	4.79	2.20-10.42	**<0.001**	2.95	0.99-8.79	**0.052**

Supplementary Table 6: Multivariable logistic regression analysis for in-hospital mortality and three-month mortality in patients without baseline renal impairment (eGFR at admission <60mL/min/1.73 m^2)

NIHSS, National Institutes of Health Stroke Scale; pmRS, premorbid modified Rankin Scale; PC-AKI, post-contrast-AKI; TICI, Thrombolysis In Cerebral Infarction; sICH, symptomatic intracerebral hemorrhage. P-values ≤0.5 are displayed in bold.
