# Supplementary material for: The Impact of Post-contrast Acute Kidney Injury on In-hospital Mortality After Endovascular Thrombectomy in Patients With Acute Ischemic Stroke
Source: Front Neurol. 2021 Jun 7;12:665614. doi: 10.3389/fneur.2021.665614 (PMC8215575; doi:10.3389/fneur.2021.665614)
Supplement: Supplementary file 7 [file Table_7.pdf]

	Unfavorable functional outcome (mRS>1), n=749 of 1169 patients			Unfavorable functional outcome (mRS>2), n=588 of 1169 patients			Early neurological deterioration (increase of NIHSS≥4 points), n=112 of 1169 patients			sICH, n=38 of 1169 patients		
	Multivariable logistic regression analyses											
	OR	95%-CI	P									
Age (per year increasing)	1.02	1.01-1.04	**0.009**	1.03	1.02-1.04	**<0.001**	1.00	0.99-1.02	0.675	0.99	0.97-1.01	0.410
NIHSS at admission (per point increasing)	1.09	1.06-1.12	**<0.001**	1.08	1.06-1.10	**<0.001**	-	-	-	1.01	0.97-1.05	0.627
Preexisting functional impairment (pmRS>1 vs. ≤1)	7.32	4.22-12.70	**<0.001**	3.46	2.45-4.88	**<0.001**	0.89	0.58-1.36	0.584	1.04	0.48-1.63	0.696
Baseline renal impairment (eGFR<60 vs. ≥60 at admission)	1.28	0.84-1.95	0.252	1.06	0.75-1.48	0.751	0.98	0.63-1.51	0.913	1.22	0.66-2.24	0.533
PC-AKI (vs. no PC-AKI)	1.64	0.47-5.77	0.440	1.57	0.52-4.72	0.427	1.34	0.45-4.02	0.602	1.50	0.34-6.56	0.594
Posterior circulation stroke (vs. anterior circulation stroke)	0.78	0.39-1.55	0.477	0.59	0.32-1.06	0.078	0.90	0.45-1.79	0.770	0.49	0.15-1.67	0.257
Failed recanalization (TICI 0-2a vs. 2b-3)	3.06	1.79-5.24	**<0.001**	4.36	2.80-6.80	**<0.001**	2.06	1.35-3.14	**0.001**	1.49	0.80-2.75	0.207
sICH (vs. no sICH)	14.90	1.89-107.94	**0.010**	7.99	2.67--23.92	**<0.001**	2.45	1.24-4.83	**0.010**	-	-	-

Supplementary Table 7: Multivariable logistic regression analysis impact of PC-AKI on functional outcome (dichotomized at a mRS scale of 1 and 2, without in-hospital death), early neurological deterioration and symptomatic intracerebral hemorrhage

NIHSS, National Institutes of Health Stroke Scale; pmRS, premorbid modified Rankin Scale; eGFR, estimated glomerular filtration rate (mL/min/1.73 m^2); PC-AKI, post-contrast-AKI; TICI, Thrombolysis In Cerebral Infarction; sICH, symptomatic intracerebral hemorrhage. P-values ≤0.5 are displayed in bold.
