# Supplementary material for: The Impact of Post-contrast Acute Kidney Injury on In-hospital Mortality After Endovascular Thrombectomy in Patients With Acute Ischemic Stroke
Source: Front Neurol. 2021 Jun 7;12:665614. doi: 10.3389/fneur.2021.665614 (PMC8215575; doi:10.3389/fneur.2021.665614)
Supplement: Supplementary file 8 [file Table_8.pdf]

	In-hospital mortality, n=127 of 1053 patients, only anterior-circulation stroke			*3-month mortality (N=138, only survivors of the acute hospital stay were considered), only anterior-circulation stroke*		
	Multivariable logistic regression analyses					
	OR	95%-CI	*P*	OR	95%-CI	*P*
Age (per year increasing)	1.04	1.02-1.06	**<0.001**	1.04	1.02-1.06	**<0.001**
NIHSS at admission (per point increasing)	1.05	1.03-1.08	**<0.001**	1.09	1.06-1.13	**<0.001**
Preexisting functional impairment (pmRS>1 vs. ≤1)	0.98	0.65-1.47	0.904	4.55	1.52-13.59	**0.007**
Baseline renal impairment (eGFR<60 vs. ≥60 at admission)	1.22	0.81-1.84	0.334	1.22	0.800-1.86	0.355
PC-AKI (vs. no PC-AKI)	2.26	0.82-6.20	0.114	2.51	1.67-3.78	**<0.001**
Failed recanalization (TICI 0-2a vs. 2b-3)	2.07	1.36-3.16	**0.001**	2.80	1.78-4.38	**<0.001**
sICH vs. no sICH	2.97	1.51-5.83	**0.002**	2.96	1.90-6.75	**0.010**

Supplementary Table 8: Multivariable logistic regression analysis for in-hospital mortality and 3-month mortality in patients with anterior circulation stroke

NIHSS, National Institutes of Health Stroke Scale; pmRS, premorbid modified Rankin Scale; eGFR, estimated glomerular filtration rate (mL/min/1.73 m^2); PC-AKI, post-contrast-AKI, TICI, Thrombolysis In Cerebral Infarction; sICH, symptomatic intracerebral hemorrhage. P-values ≤0.5 are displayed in bold.
